# Machine‐learning based radiogenomics analysis of MRI features and metagenes in glioblastoma multiforme patients with different survival time

**DOI:** 10.1111/jcmm.14328

**Published:** 2019-04-18

**Authors:** Xin Liao, Bo Cai, Bin Tian, Yilin Luo, Wen Song, Yinglong Li

**Affiliations:** ^1^ Department of Medical Imaging The Affiliated Hospital of Guizhou Medical University Guiyang Guizhou China; ^2^ Department of Medical Imaging The Third People's Hospital of Guizhou Province Guiyang Guizhou China; ^3^ Department of Interventional Radiology Guizhou Provincial People's Hospital Guiyang Guizhou China

**Keywords:** death day to diagnosis, EREG, glioblastoma multiforme, machine learning, radiogenomics, ROS1, TIMP1

## Abstract

**Background:**

This study aimed to examine multi‐dimensional MRI features’ predictability on survival outcome and associations with differentially expressed Genes (RNA Sequencing) in groups of glioblastoma multiforme (GBM) patients.

**Methods:**

Radiomics features were extracted from segmented lesions of T2‐FLAIR MRI data of 137 GBM patients. Radiomics features include intensity, shape and textural features in seven classes were included in the analysis. Patients were divided into two groups depending on their survival time (shorter or longer than 1‐year survival). Four different machine learning algorithms were implemented to construct the prediction models. Features with top importance (importance >0.04) were selected to construct the prediction model using the model with the best performance. The interactions between image features and genomics were then analysed with Pearson's correlation analysis.

**Results:**

The GBDT model with 72 features with highest importance had the highest accuracy of 0.81 on both short and long survival time classes, and the area under the curve (AUC) of the receiver operative characteristic (ROC) of the short and long survival time class were 0.79 and 0.81. Six metagenes showed significant interactive effect (*P* < 0.05), and Pearson's correlation analysis revealed that three of these metagenes (*TIMP1*,*ROS1 EREG*) showed moderate (0.3 < |*r*| < 0.5) or high correlation (|*r*| > 0.5) with image features.

**Conclusion:**

Radiogenomics analysis shows that MRI features are predictive of survival outcomes, and image features are highly associated with selective metagenes. Radiogenomics analysis is a useful method for optimizing clinical diagnosis and selecting effective treatments.

## 1 INTRODUCTION

Glioblastoma multiforme (GBM), one of the most invasive and fatal brain tumours that develops from glial cells, can severely affects the central nervous system and general health [1]. The percent 5‐year surviving rate was estimated to be 33.2% between 2008 and 2014 according to statistics from the Surveillance, Epidemiology and End Results (SEER) database and the Centers for Disease Control and Prevention's National Center for Health Statistics (https://seer.cancer.gov/csr/1975_2015/). Due to the heterogeneous nature of GBM, relatively high age of disease onset, migration of malignant cells to surrounding tissue, the treatment outcome for GBM are highly variable, yielding an average survival rate of 12.6 months [2]. Current clinical practice for treating GBM mostly involves tumour resection and chemotherapy [3].

Genomics study is an essential method to study GBM by examining alternations in genomic pathways and identifying relevant biomarkers. Gene studies involving tissues, plasma, or cell lines used protein expression data to reveal that common alternations in GBM include mutations of specific gene and proteins such as RTKs, TP53 RB1 and increased expression of EGFR and PDGFRA [4, 5]. However, tissue sample is usually acquired after biopsy and may not be suitable for all patients, especially for early diagnosis.

Neuroimaging of GBM is a non‐invasive tool for disease diagnosis and monitor treatment outcome. A wide range of MR techniques including T1, T2 and FLAIR imaging are used to capture GBM characteristics. Typically, GBM appears as a heterogeneous enhancement region with a non‐enhancing necrosis in the center [6]. FLAIR sequences have advantages of showing abnormalities more clearly [7]. MRI‐based features were shown to be highly predictive of tumour grading in GBM [8]. Textural image features were associated with CD3 T cell infiltration status in GMB [9].

In recent years, the emergence of radiogenomics, combing radiomics image features and genomics, allows the study of GBM more comprehensively. For example, MRI parameters revealed that haemodynamic abnormalities were associated with the expression level of the mTOR‐EGFR pathway in [10]. Based on previous findings, we aimed to investigate the machine learning based methods in combination with radiogenomics to study the associations among MRI features, genomics and the survival rates in GBM patients. Computer assisted methods allow more comprehensive characterization of imaging data and more sophisticated way to predict disease outcome. We hypothesize that radiomics features of FLAIR imaging data can be predictive of patients’ survival, and radiogenomics analysis can reveal the linkage between images features and known genes in previously defined molecular pathways.

## 2 MATERIALS AND METHODS

### 2.1 Dataset

**Figure 1:**
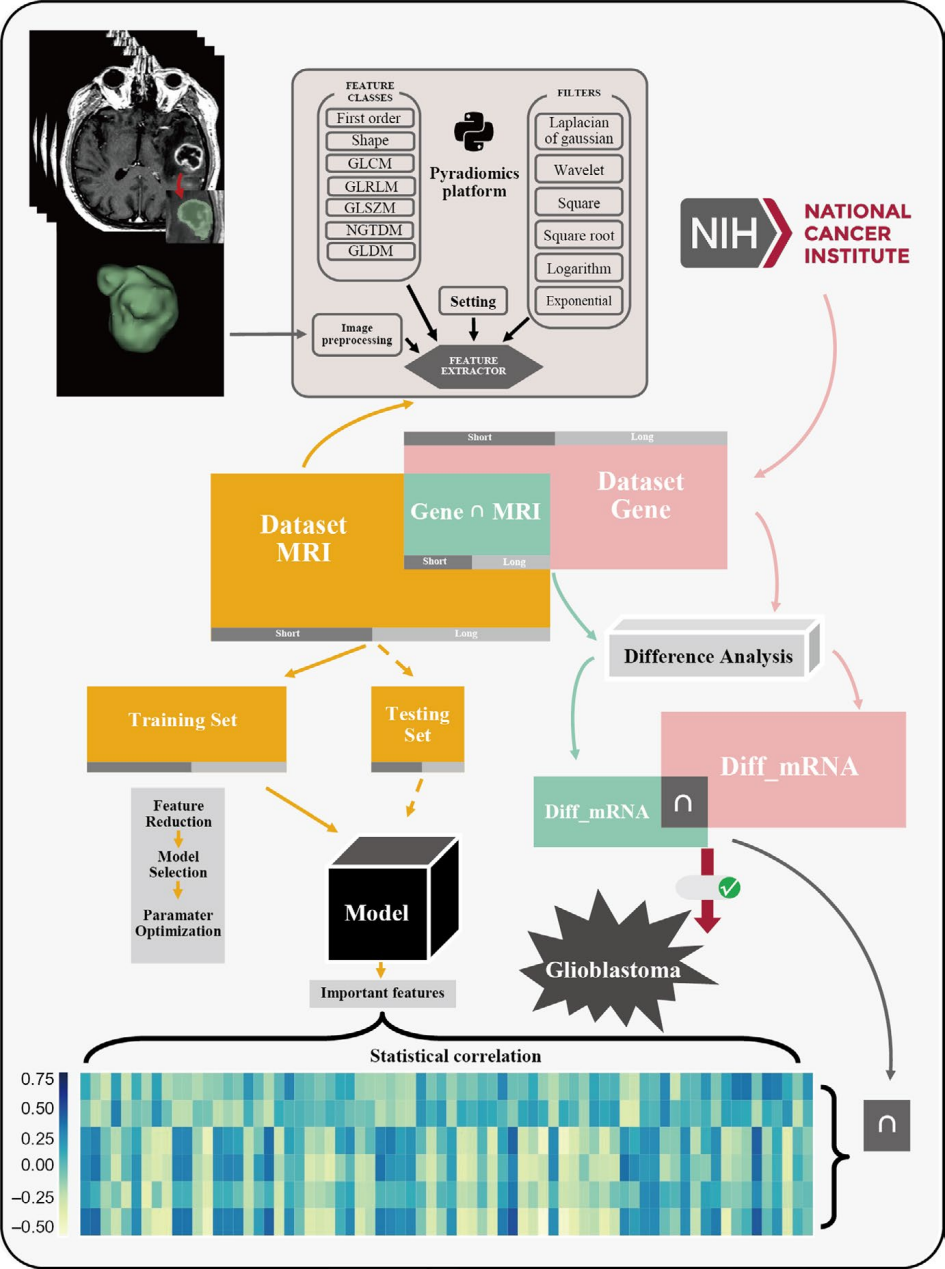
The workflow of this study. The radiomics workflow. Lesions were segmented from untreated MR images. Feature extraction was performed from lesions by pyradiomics. The radiomics features were selected for classifier model constructing. And the classifier model was evaluated by confusion matrix and ROC curves

MRI data were obtained from the Cancer Imaging Archive (TCIA) (https://wiki.cancerimagingarchive.net/display/Public/TCGA-GBM), and corresponding genomics data were acquired from the Genomic Data Commons (GDC) Data Portal. A total of 137 patients with MRI data, 129 patients with known genomic data were included in the analysis and 46 patients were the intersection of MRI data set and gene data set. Patient characteristics are summarized in Table 1. Because the average survival rate of GBM patients was reported to be 12.6 months [2], and all the patients in our cohort has demised during follow‐up, for the classification purpose, we used 1 year as a threshold and the patients were divided into short (<1 year) and long (>1 year) survival groups. Figure 1 shows the process of the workflow of this study.

**Table 1:** Clinical characteristics of the cohort. This table shows the clinical information of the data analysed in this study. Gene∩MRI means that the dataset has both genetic data and MRI data

	Gender		Death days to diagnosis		Number	Age	
	Men	Female	Long (>1 year)	Short (<1 year)	Total	Mean	SD
Gene	85	44	68	61	129	62.05 (25‐89)	12.55
MRI	85	52	71	66	137	61.24 (16‐86)	13.53
Gene+MRI	27	19	25	21	46	61.86 (32‐86)	12.04

### 2.2 Image preprocessing and lesion segmentation

Lesion segmentation is required before feature extraction. Lesion volumes were manually delineated by an experienced radiologist using 3D slicer (https://www.slicer.org/). All original loaded MRI images of patients were DICOM format. After adding MRI data into 3D slicer, we selected the Segment Editor module to segment the lesion.

### 2.3 Feature extraction

Feature extraction was performed using a Python software package Pyradiomics [11]. First‐order and multi‐dimensional features were extracted from seven feature classes including First Order Features, Shape Features, Gray Level Co‐occurrence Matrix (GLCM) Features, Gray Level Size Zone Matrix (GLSZM) Features, Gray Level Run Length Matrix (GLRLM) Features, Neighboring Gray Tone Difference Matrix (NGTDM) Features, Gray Level Dependence Matrix (GLDM) Features. Detailed number of each feature is listed in Table S1.

### 2.4 Machine learning model construction and evaluation

The MRI dataset was divided into the training and testing sets according to a ratio of 7:3. Four machine learning algorithms including GBDT (Gradient Boosting Decision Tree), logistic regression, support vector machine (SVM) and KNN (k‐nearest neighbours) were tested. These four methods are representative in their own category. Gradient boosting decision tree is a tree‐based ensemble machine learning model which can achieve state‐of‐the‐art accuracy in classification and regression. Logistic regression is a classic probabilistic model. Support vector machine is another widely used model featured by kernel trick [12]. As for k‐nearest neighbours, it is a typical lazy‐learning method and is frequently treated as a benchmark in predictive modelling [13]. Feature importance was computed using GBDT (https://doi.org/10.2307/2699986), implemented by python package sci‐kit learn (https://scikit-learn.org/stable/index.html). In the final prediction model construction, feature with importance value smaller than 0.04 which were treated as not important were excluded. This threshold is chosen after the manually checking of the distribution of feature importance.

Confusion matrices and receiver operative characteristic (ROC) were computed to evaluate and compare the performances of all four machine learning models. The model that is most predictive of GBM patients’ survival time is chosen for further radiogenomics analysis.

### 2.5 Relevant gene selection

Differentially expressed genes (DEGs) analysis was performed with R software, using package DESeq2. A gene is declared to be DEGs if a difference or change observed in read counts or expression is statistically significant. Fold change and *t* test are widely used methods to estimate gene variances in practice [14]. The condition we added for screening out DEGs was |log_2_(fold change)| > 1 and adjusted *P* < 0.05. And the same DEGs analytical process was applied to Dataset of Gene and Dataset with both MRI and Gene data to obtain DEGs. DEGs are treated as metagenes in our analysis.

After screening out DEGs, the number of samples was reduced while individual differences among groups were enhanced. In order to screen for efficiently DEGs, we selected the DEGs from the intersection of the Genetic Dataset and Dataset which contain both MRI and Gene data.

### 2.6 Correlations between image features and genomics

To survey the potential correlations between the important image features of the classification model and the efficiently DEGs, we performed Pearson correlation analysis. Statistically, the absolute value of Pearson's correlation coefficient is between 0.3 and 0.5, indicating a moderate correlation and greater than 0.5 indicating a significant correlation. We also filtered out the weak correlation based on Benjamin‐Hochberg adjusted P‐values [15]. For the generalization purpose, we used the Pearson correlation as the final correlation selection metrics.

### 2.7 Risk stratification of metagenes

In order to survey the prognostic power of identified metagenes. We used the maximally selected rank statistics [16], implemented by R package maxstat to find the optimal cut point for the risk stratification on the basis of expression value of corresponding metagenes. Afterwards, we used Kaplan‐Meier (KM) estimator to measure the patients’ survival rates in high and low gene expression strata and plotted the aforementioned information by R package survminer.

## 3 RESULTS

### 3.1 Selected radiomics features

Thresholding based on feature importance (importance index >0.04) resulted in a total of 72 features for constructing the final prediction model. The threshold is chosen after the manually checking of feature importance distribution (Figure S1A). Table 2 lists the full name and abbreviation of the corresponding 72 features in the model.

**Table 2:** Detailed names and abbreviations of 72 features

Full name	Short name
log‐sigma‐3‐0‐mm‐3D_gldm_SmallDependenceEmphasis	gldm‐SDE
wavelet‐HHL_gldm_DependenceNonUniformityNormalized	gldm‐DNUN
log‐sigma‐3‐0‐mm‐3D_firstorder_Uniformity	firstorder‐Uniformity
wavelet‐HHH_glszm_GrayLevelNonUniformityNormalized	glszm‐GLNUN
wavelet‐LLL_glcm_InverseVariance	glcm‐IV
wavelet‐LLH_glszm_ZonePercentage	glszm‐ZP
wavelet‐LLH_glcm_Idm	glcm‐LLH‐Idm
wavelet‐HLH_glcm_InverseVariance	glcm‐HLH‐IV
log‐sigma‐4‐0‐mm‐3D_glcm_Idm	glcm‐Idm
wavelet‐HLH_glcm_SumSquares	glcm‐HLH‐SS
wavelet‐HLH_gldm_GrayLevelVariance	gldm‐HLH‐GLV
wavelet‐LLL_glszm_ZoneVariance	glszm‐LLL‐ZV
log‐sigma‐4‐0‐mm‐3D_glcm_Id	glcm‐Id
wavelet‐HHL_glrlm_RunLengthNonUniformityNormalized	glrlm‐HHL‐RLNUN
wavelet‐HLL_glrlm_RunLengthNonUniformityNormalized	glrlm‐HLL‐RLNUN
wavelet‐HLH_glszm_SmallAreaEmphasis	glszm‐HLH‐SAE
wavelet‐LLL_glcm_Correlation	glcm‐LLL‐Correlation
wavelet‐HHH_glszm_SmallAreaEmphasis	glszm‐SAE
wavelet‐HHL_glcm_DifferenceAverage	glcm‐DA
log‐sigma‐5‐0‐mm‐3D_glcm_Correlation	glcm‐Correlation
log‐sigma‐4‐0‐mm‐3D_glrlm_ShortRunEmphasis	glrlm‐SRE
original_glrlm_RunLengthNonUniformityNormalized	glrlm‐RLNUN
wavelet‐LHL_glcm_Idn	glcm‐LHL‐Idn
wavelet‐HLH_glcm_Idn	glcm‐HLH‐Idn
wavelet‐HHL_glcm_Idn	glcm‐HHL‐Idn
wavelet‐LLL_glrlm_RunLengthNonUniformityNormalized	glrlm‐LLL‐RLNUN
wavelet‐LLL_glcm_Imc2	glcm‐LLL‐Imc2
log‐sigma‐5‐0‐mm‐3D_glcm_Idn	glcm‐Idn
log‐sigma‐4‐0‐mm‐3D_glcm_Idmn	glcm‐Idmn
wavelet‐HLH_glcm_ClusterProminence	glcm‐HLH‐CP
wavelet‐HHL_glcm_DifferenceEntropy	glcm‐HHL‐DE
wavelet‐HHH_firstorder_InterquartileRange	firstorder‐HHH‐IR
wavelet‐HHL_firstorder_InterquartileRange	firstorder‐HHL‐IR
log‐sigma‐3‐0‐mm‐3D_firstorder_Entropy	firstorder‐Entropy
wavelet‐LLH_gldm_LargeDependenceEmphasis	gldm‐LLH‐LDE
wavelet‐LLH_glcm_DifferenceEntropy	glcm‐LLH‐DE
wavelet‐HLH_firstorder_InterquartileRange	firstorder‐HLH‐IR
wavelet‐LHL_gldm_LargeDependenceEmphasis	gldm‐LHL‐LDE
original_gldm_LargeDependenceEmphasis	gldm‐LDE
wavelet‐LHH_glcm_SumEntropy	glcm‐LHH‐SE
wavelet‐LHH_glszm_LargeAreaEmphasis	glszm‐LHH‐LAE
log‐sigma‐5‐0‐mm‐3D_glcm_SumSquares	glcm‐SS
log‐sigma‐2‐0‐mm‐3D_glcm_Contrast	glcm‐Contrast
wavelet‐LHH_gldm_LargeDependenceEmphasis	gldm‐LHH‐LDE
log‐sigma‐5‐0‐mm‐3D_glrlm_RunEntropy	glrlm‐RE
log‐sigma‐5‐0‐mm‐3D_glszm_ZoneVariance	glszm‐ZV
wavelet‐HHL_glcm_JointEntropy	glcm‐JointEntropy
log‐sigma‐3‐0‐mm‐3D_glszm_LargeAreaEmphasis	glszm‐LAE
wavelet‐LLH_gldm_GrayLevelNonUniformity	gldm‐GLNU
wavelet‐HLL_glszm_GrayLevelNonUniformity	glszm‐GLNU
log‐sigma‐5‐0‐mm‐3D_glszm_ZoneEntropy	glszm‐ZE
wavelet‐HLL_gldm_GrayLevelNonUniformity	gldm‐HLL‐GLNU
wavelet‐LLL_glcm_SumEntropy	glcm‐LLL‐SE
wavelet‐HHH_glrlm_HighGrayLevelRunEmphasis	glrlm‐HHH‐HGLRE
wavelet‐HHH_firstorder_Maximum	firstorder‐Max
wavelet‐LLH_gldm_GrayLevelVariance	gldm‐LLH‐GLV
wavelet‐LLH_glcm_SumSquares	glcm‐LLH‐SS
original_firstorder_MeanAbsoluteDeviation	firstorder‐MAD
wavelet‐LLH_glcm_JointAverage	glcm‐JointAverage
wavelet‐LLH_glrlm_GrayLevelVariance	glrlm‐GLV
log‐sigma‐2‐0‐mm‐3D_gldm_DependenceNonUniformity	gldm‐DNU
log‐sigma‐5‐0‐mm‐3D_glrlm_HighGrayLevelRunEmphasis	glrlm‐HGLRE
wavelet‐HLL_firstorder_Variance	firstorder‐Variance
wavelet‐LHL_firstorder_RootMeanSquared	firstorder‐RMS
original_glrlm_ShortRunHighGrayLevelEmphasis	glrlm‐SRHGLE
original_glszm_SmallAreaHighGrayLevelEmphasis	glszm‐SAHGLE
log‐sigma‐2‐0‐mm‐3D_glcm_ClusterProminence	glcm‐CP
original_glszm_HighGrayLevelZoneEmphasis	glszm‐HGLZE
wavelet‐LLL_gldm_SmallDependenceHighGrayLevelEmphasis	gldm‐SDHGLE
original_glszm_LargeAreaHighGrayLevelEmphasis	glszm‐LAHGLE
log‐sigma‐2‐0‐mm‐3D_gldm_LargeDependenceHighGrayLevelEmphasis	gldm‐LDHGLE
wavelet‐LLL_gldm_LargeDependenceHighGrayLevelEmphasis	gldm‐LLL‐LDHGLE

### 3.2 Model comparison

**Figure 2:**
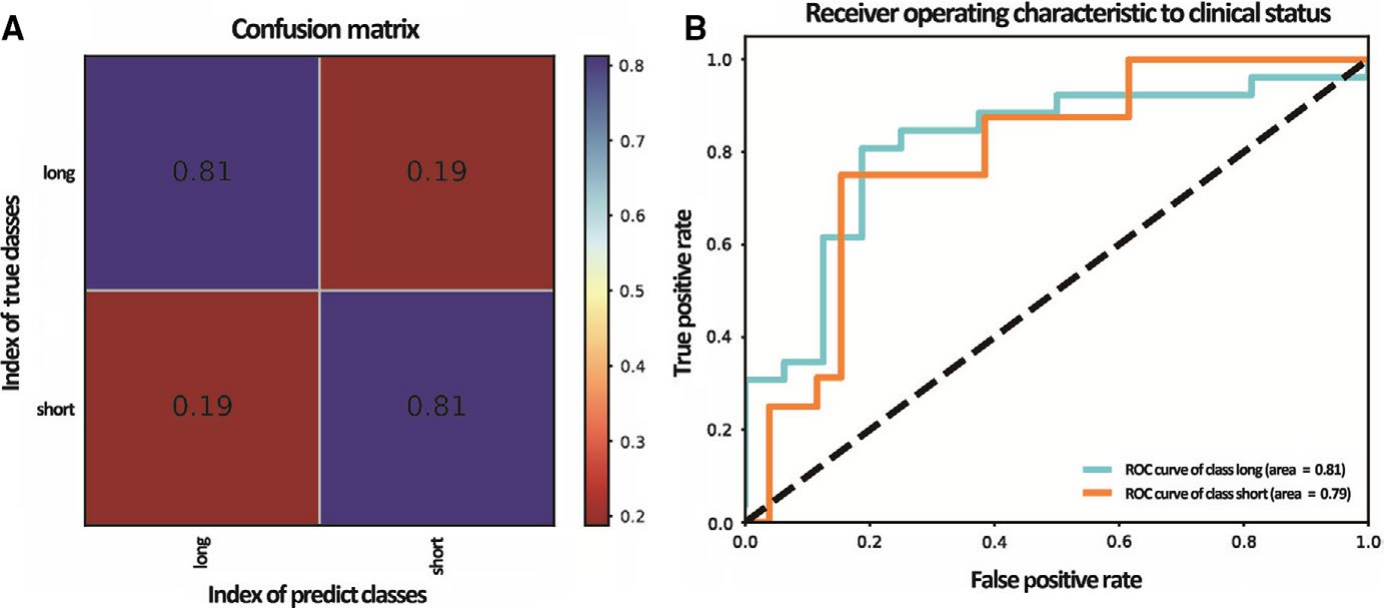
The performance of the GBDT classifier. A, Confusion matrix (The horizontal line means the number of predicted in each group; the vertical line means the actual number of each group. The leading diagonal represents correct prediction; the minor diagonal represents incorrect prediction). B, Receiver operating characteristic (ROC) curve. (*X* axis represents false positive rate and *Y* axis is true positive rate.)

Among the four machine learning algorithms, GBDT had the highest accuracy of 0.81 for discriminating patients with short or long survival in testing set, while the accuracy of logistic regression, SVM and KNN is 0.69, 0.76 and 0.79, respectively. Figure 2 shows the performance of the GBDT classifier. Figure 2A is the confusion matrix demonstrating the proportion of correct and wrong predictions in each survival class. Figure 2B shows the ROC curves for predicting patients with short and long survivals, yielding an AUC value of 0.79 for short‐survival class and 0.81 for long‐survival class.

### 3.3 Metagenes selection

**Figure 3:**
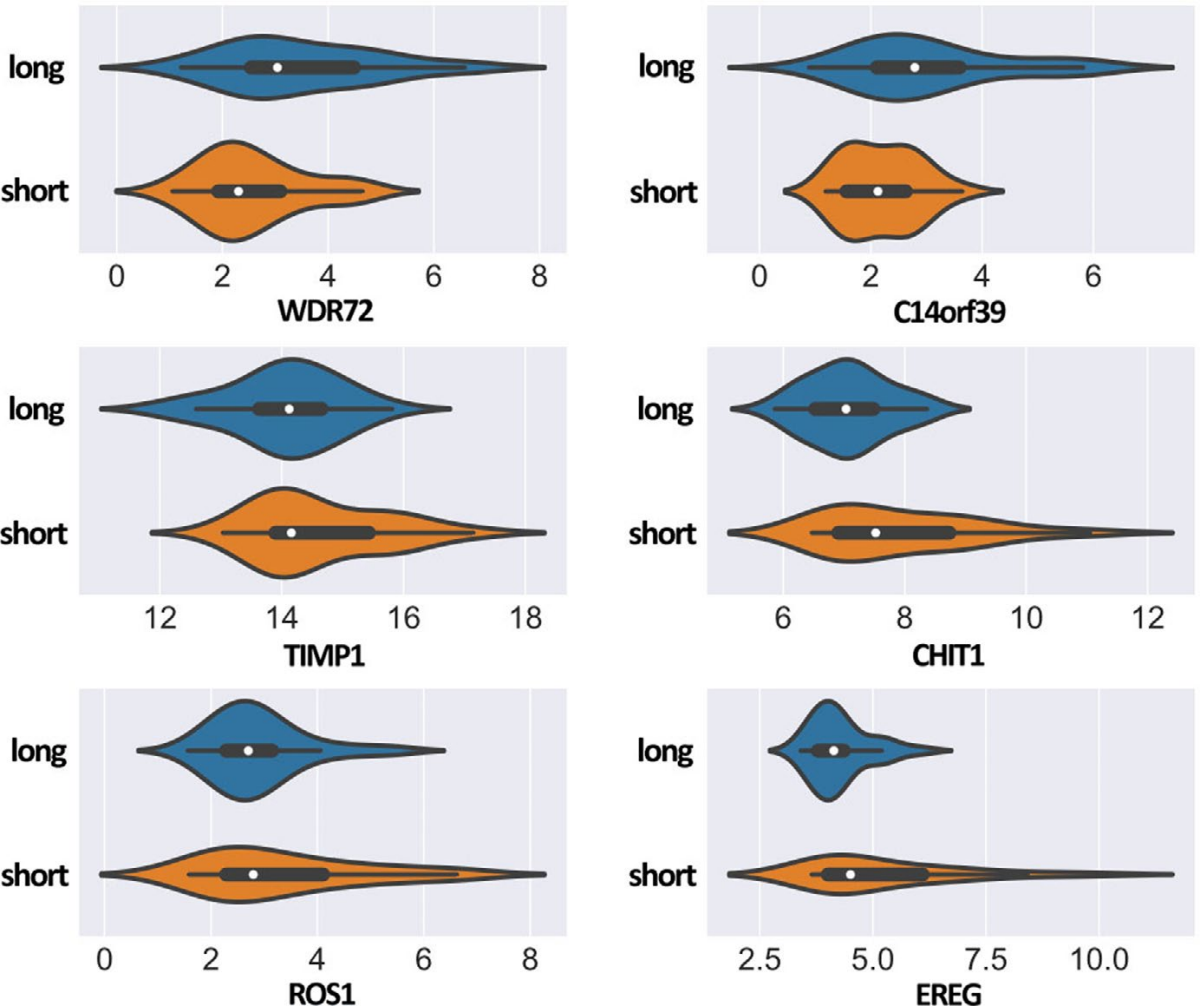
Gene expressions of six gene. The distribution of six Gene expressions among patients with short vs. long survival time. The expression levels of six genes were significantly different in two classes of survival patients

Six metagenes including *WDR72*,* C14orf39*,* TIMP1*,* CHIT1*,* ROS1* and *EREG* were found to have significantly different expression levels among patients with short vs. long survival time (Figure 3). The difference analysis of these six genes was conducted between the long and short group, and the result is shown in Table 3.

**Table 3:** Intersection of difference analysis between group long and short. Threshold of difference analysis adjusted *P* < 0.05 & |log2FoldChange|>1

mRNA	Base mean	log_2_FC	*P* value	*P*.adj	Base mean	log_2_FC	*P* value	*P*.adj
WDR72	22.54202	−1.53057	0.00001	0.00327	22.10411	−2.66113	<0.00001	0.00678
C14orf39	9.74465	−1.03545	0.00051	0.04247	13.31277	−2.21750	0.00002	0.02407
TIMP1	25087.60318	1.06274	<0.00001	0.00109	28495.57870	1.53657	0.00004	0.03445
CHIT1	345.04844	1.40483	<0.00001	0.00109	329.45162	2.05879	0.00001	0.02339
ROS1	16.00196	1.42552	<0.00001	0.00178	21.31838	2.24119	0.00003	0.03445
EREG	57.47073	2.63671	<0.00001	<0.00001	69.45137	2.75592	0.00002	0.00678

FC: fold change; p.adj: adjusted p value.

### 3.4 Relationship between genes and image features

**Figure 4:**
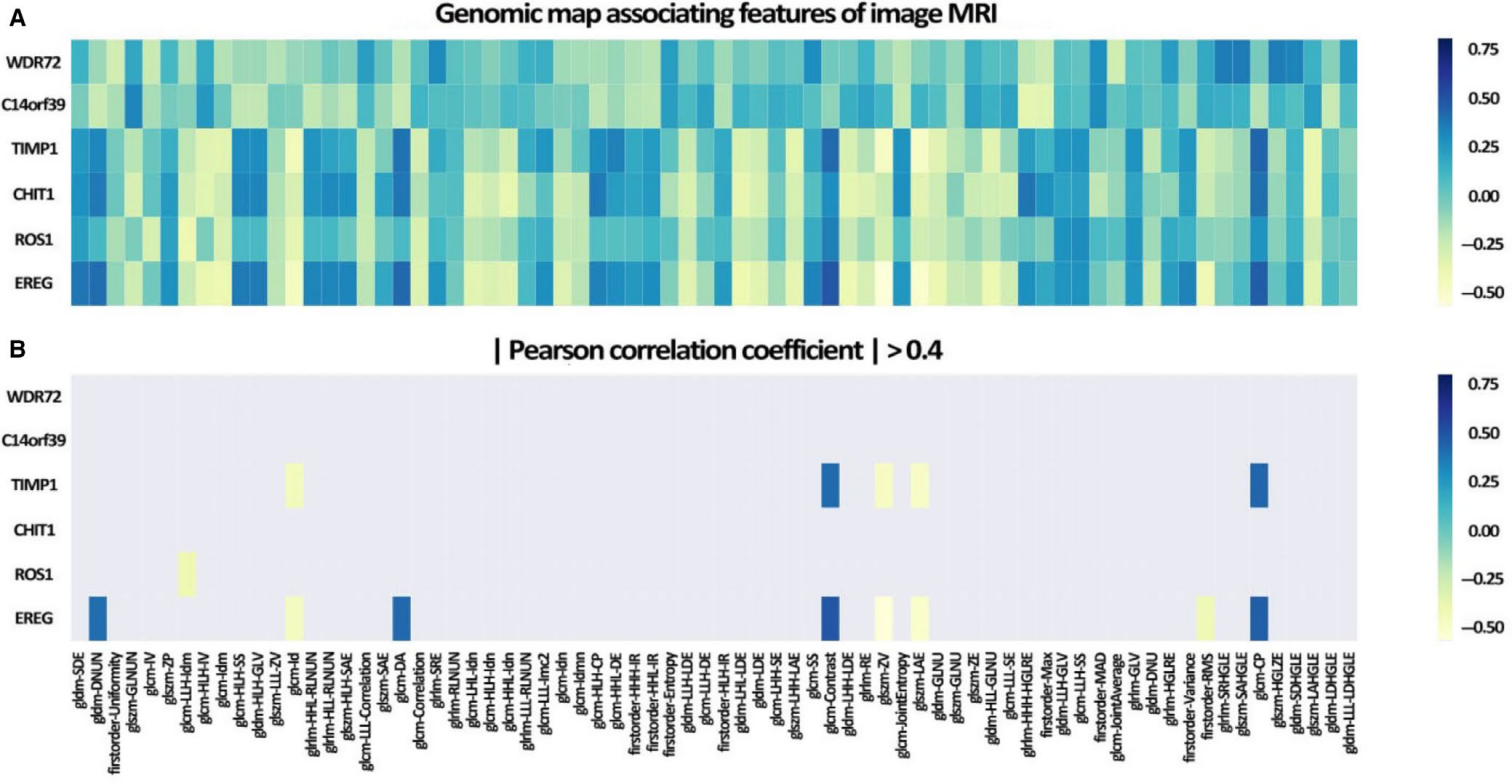
Correlation between genes and image features. The matrix correlation between top image features and genes. A, The matrix showing the correlations between top image features and genes. B, The correlations between top image features and genes after the threshold of 0.4 was applied to filter out features that had weak correlations with corresponding genes

**Figure 5:**
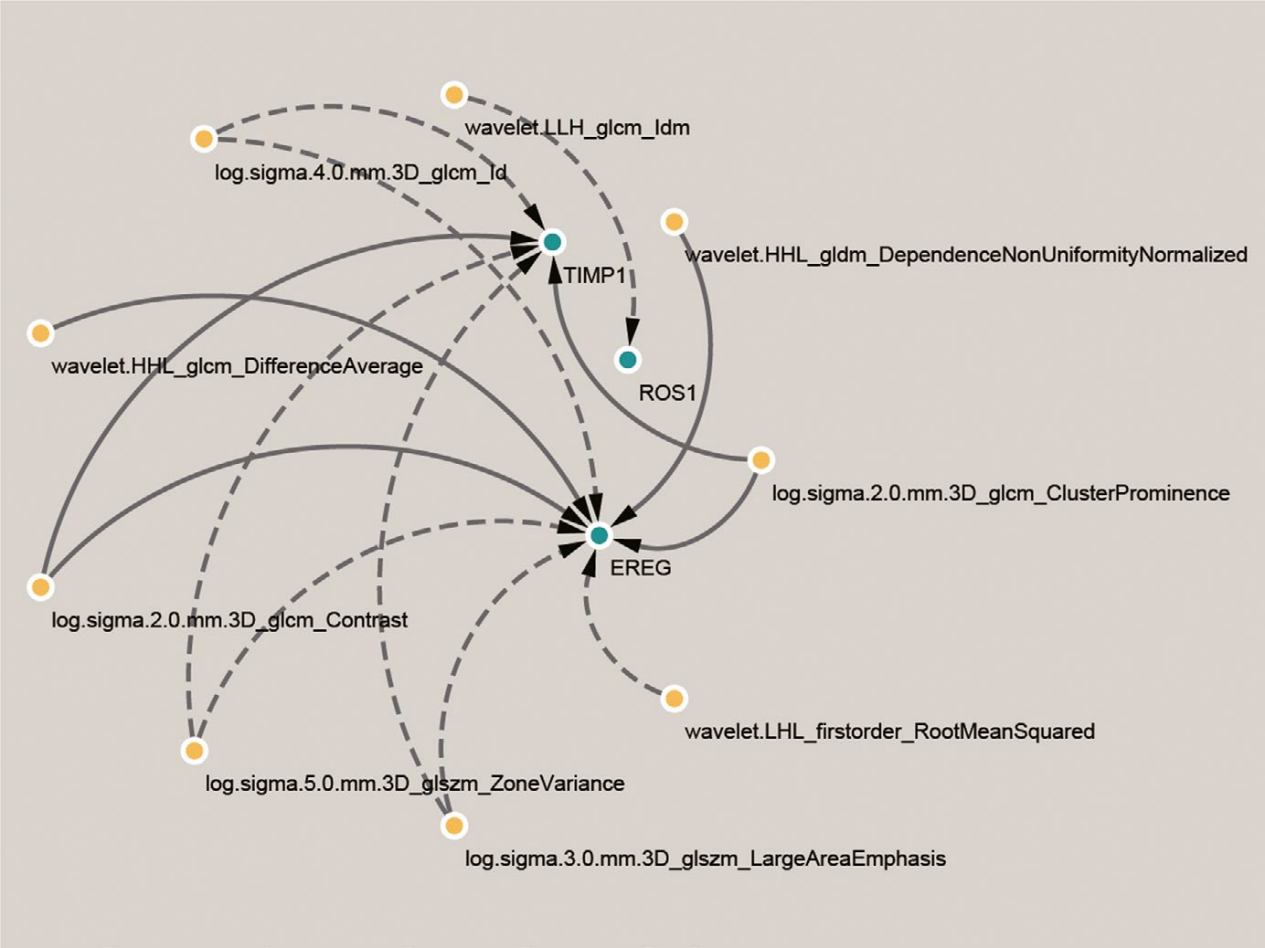
Correlation between three genes and nine image features. The correlations of nine image features and three genes. The solid line represents a positive correlation, and the dotted line represents a negative correlation

Figure 4A is the matrix showing the correlations between top image features and metagenes. A threshold of 0.4 was applied to filter out features that had weak correlations with corresponding metagenes (Figure 4B). A total of nine image features (including eight textural features and one intensity‐based feature) were strongly correlated with three metagenes (*TIMP1*,* ROS1*,* EREG*). *EREG* is positively associated with Dependence Non‐Uniformity (gldm‐DNUN), Difference Average (glcm‐DA), Contrast (glcm‐Contrast) and Cluster Prominence (glcm‐CP) and negatively associated with Inverse Difference (glcm‐Id), Zone Variance (glszm‐ZV), LargeArea Emphasis (glszm‐LAE) and Root Mean Squared (firstorder‐RMS). *ROS1* gene is negatively associated with Inverse Difference Moment (glcm‐LLH‐Idm). *TIMP1* is positively associated with Contrast (glcm‐Contrast), Cluster Prominence (glcm‐CP) and negatively associated with Inverse Difference (glcm‐Id), Zone Variance (glszm‐ZV), LargeArea Emphasis (glszm‐LAE). Correlation thresholding based on Benjamini‐Hochberg adjusted P‐values was show in Figure S1B. The correlations of image features and metagenes are shown in Figure 5.

## 4 DISCUSSION

### 4.1 Associations between image features and survival outcome

Our results indicate that prediction models using radiomics features can discriminate patients with under or over 1‐year survival time, suggesting that MR image features are predictive of survival outcome in GBM. Textual features such as large dependence emphasis and entropy are especially indicative of clinical outcome. Similarly, Gutman et al. showed that contrast‐enhanced tumour volume was strongly correlated with poor survival [17]. Lao *et al*. used deep learning method to correlate radiomics features with survival in GBM [18]. Our study provides additional evidence of using computer assisted learning methods to examine the relevant information contained in image features. Compared to conventional manual analysis approaches, radiomics analysis can have the advantage of providing more efficient and unbiased quantification.

### 4.2 Differentially expressed genes in different survival groups

**Figure 6:**
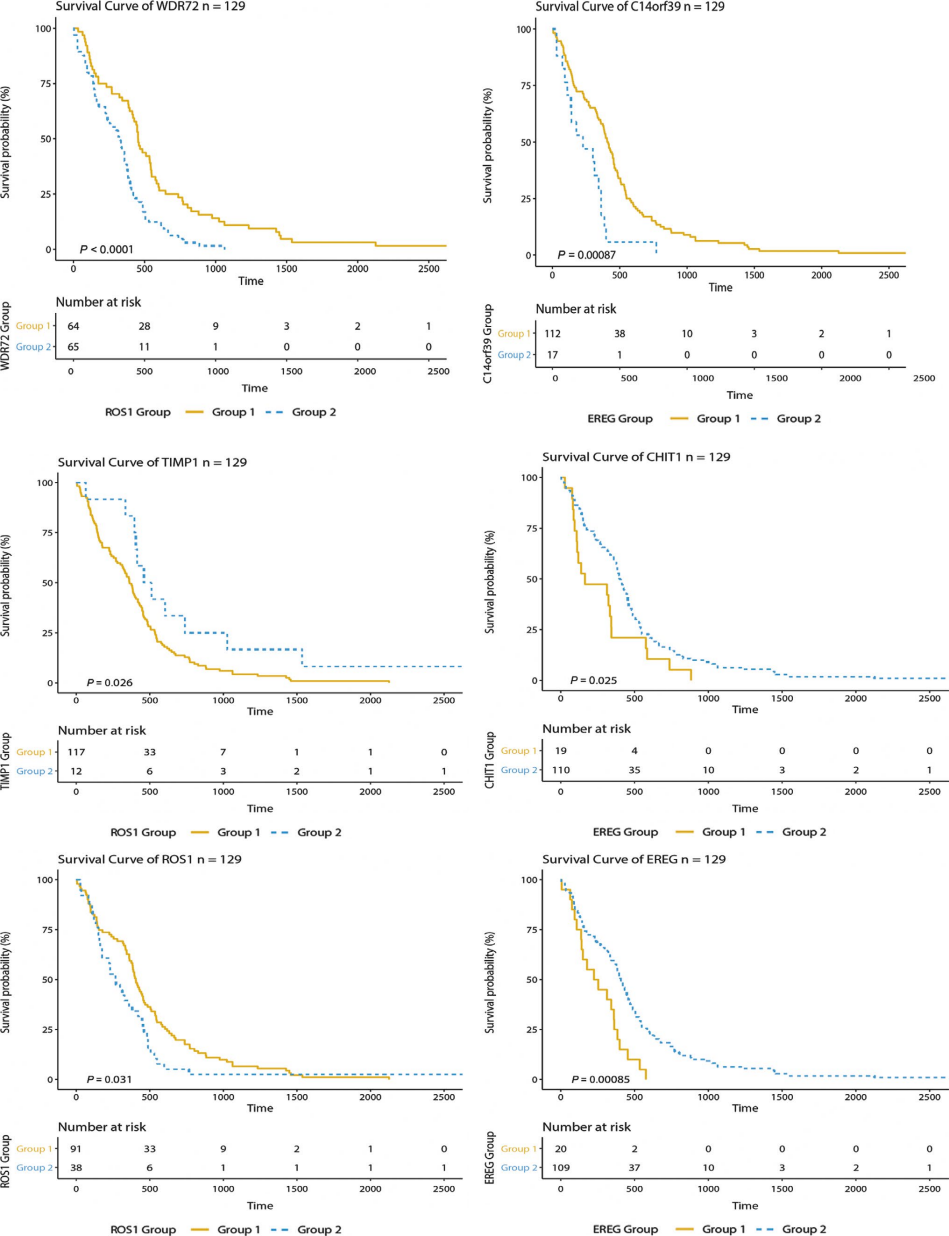
The Kaplan‐Meier survival curve of six genes. KM survival curves show significant overall survival differences between higher‐expression levels and lower‐expression levels of survival rates of patients. For all the subplots, the ‘group 1’, coloured by yellow, stands for higher‐expression group at the optimal cut point identified by maximally selected rank statistics

We identified six genes (*WDR72*,* C14orf39*,* TIMP1*,* CHIT1*,* ROS1* and *EREG*) with significantly different levels of expression between short and long survival groups. To reveal the relationship between expression levels of six genes and the prognosis of patients, a survival analysis was performed. In this study, we used Kaplan‐Meier (KM) estimator to measure the patients’ survival rates in high and low gene expression [19]. Figure 6 shows the KM survival curve for six genes. The KM survival curves showed significant differences in overall survival between patients with high and low expression levels of six genes. The association between six genes expression levels and patient survival was significantly (*P* < 0.05). The C‐index of the six genes (*WDR72*,* C14orf39*,* TIMP1*,* CHIT1*,* ROS1* and *EREG*) is 0.59, 0.55, 0.47, 0.46, 0.55, 0.45, respectively. *EGFR* has long been identified as an important therapeutic target for the treatment of GBM, and in patients with low overall survival time, elevated levels of *EREG* expression has been found. [20]. *EREG* can initiate the signalling cascade, and in gastric, *EREG* is up‐regulate [21]. Previous studies have shown the Epiregulin (*EGFR*) ligands have the effect of stabilizing receptors, affecting breast cancer cells associated with differentiation function [22]. Altered *TIMP‐1* expression has been identified as a biomarker in GBM, with decreased TIMP‐1 linking to longer survival in GBM [23]. *ROS1*, which belongs to one subfamily of kinase insulin receptor genes, is a proto‐oncogene, highly expressed in a variety of tumour cells. This gene is often altered in lung cancer, of which the effects on the progression of GBM are remains to be eliminated [24].

### 4.3 Associations between image features and genes

Associating genes and microRNAs with high FLAIR volumes enables researchers to screen for molecular cancer subtypes and genomic relationship of cellular invasion. [25]. We found *TIMP‐1* and *EREG* showed similar correlations with textural features (Table 4). Similar to our finding about *EREG*, Hu *et al*. indicated six genes including EGFR were significantly correlated with imaging features in GBM [26]. Grossmann et al. showed that volumetric image features were associated with homoeostasis and cell cycling pathways, concluding that oedema in FLAIR images were most predictive of GBM subtypes and overall survival [27]. Other relevant gene, such as POSTN, was found to play important roles in the regulatory pathways through radiogenomics analysis [25].

**Table 4:** Associations between image features and metagenes. This table shows the associations between nine image features and three metagenes, and the last column is the values of Pearson correlation coefficient

Efficient DEGs	Important image features	PCC
EREG	wavelet‐HHL_gldm_DependenceNonUniformityNormalized	0.41
EREG	log‐sigma‐4‐0‐mm‐3D_glcm_Id	−0.46
EREG	wavelet‐HHL_glcm_DifferenceAverage	0.42
EREG	log‐sigma‐2‐0‐mm‐3D_glcm_Contrast	0.49
EREG	log‐sigma‐5‐0‐mm‐3D_glszm_ZoneVariance	−0.56
EREG	log‐sigma‐3‐0‐mm‐3D_glszm_LargeAreaEmphasis	−0.51
EREG	wavelet‐LHL_firstorder_RootMeanSquared	−0.41
EREG	log‐sigma‐2‐0‐mm‐3D_glcm_ClusterProminence	0.46
TIMP1	log‐sigma‐4‐0‐mm‐3D_glcm_Id	−0.43
TIMP1	log‐sigma‐2‐0‐mm‐3D_glcm_Contrast	0.42
TIMP1	log‐sigma‐5‐0‐mm‐3D_glszm_ZoneVariance	−0.47
TIMP1	log‐sigma‐3‐0‐mm‐3D_glszm_LargeAreaEmphasis	−0.49
TIMP1	log‐sigma‐2‐0‐mm‐3D_glcm_ClusterProminence	0.43
ROS1	wavelet.LLH_glcm_Idm	−0.40

DEG: differentially expressed genes; PCC: Pearson correlation coefficient.

### 4.4 Limitations and suggestions

In this study, we used MRI data of 137 to identify radiomics features, but only a subpopulation of them (46) are provided with genomics data as well. For future analysis, larger patient sample size with both imaging and genomics data may be better to detect more correlating genes. In addition to FLAIR data, additional sequences and imaging modalities can be combined for multimodal analysis, which can provide comparison results about different methods.

We selected 72 features to construct the prediction model. More advanced dimensionality reduction method can be implemented for potential improvements of dimensionality reduction and improving classification performances.

Our study validates the method of radiogenomics analysis to study the correlations among gene variables, imaging features and survival outcome in GBM. Our findings provide useful information for further examination of corresponding genes, which may potentially serve as biomarkers for GMB diagnosis and treatment indicators.

## CONFLICT OF INTEREST

The authors declare that they have no conflict of interest with the contents of this article.

## AUTHOR CONTRIBUTION

XL performed the research and wrote the paper, BC designed the research study and wrote the paper, YL(Luo) and WS analysed the data, YL(Li) contributed essential reagents and analysed the data.
