# Supplementary figures and images for: Machine‐learning based radiogenomics analysis of MRI features and metagenes in glioblastoma multiforme patients with different survival time
Source: J Cell Mol Med. 2019 Apr 18;23(6):4375–85. doi: 10.1111/jcmm.14328 (PMC6533509; doi:10.1111/jcmm.14328)

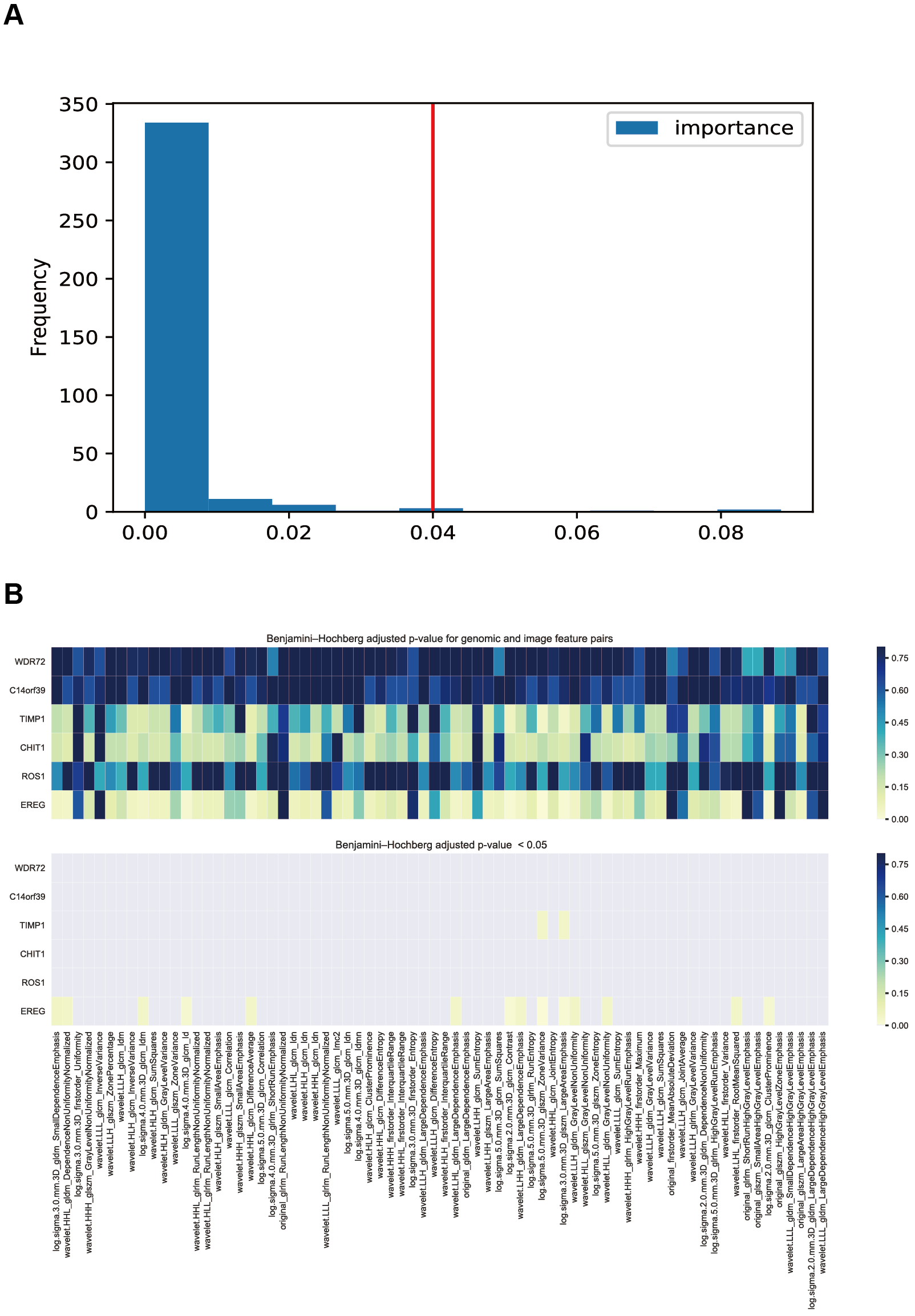

Supplement: Supplementary file 1 [file JCMM-23-4375-s001.tif]
