# Supplementary material for: Machine‐learning based radiogenomics analysis of MRI features and metagenes in glioblastoma multiforme patients with different survival time
Source: J Cell Mol Med. 2019 Apr 18;23(6):4375–85. doi: 10.1111/jcmm.14328 (PMC6533509; doi:10.1111/jcmm.14328)
Supplement: Supplementary file 2 [file JCMM-23-4375-s002.docx]

**Table S1. Number of Features in Each Type**

| Feature Type | Number |
| --- | --- |
| First Order | 145 |
| Shape | 13 |
| GLCM | 139 |
| GLSZM | 171 |
| GLRLM | 170 |
| GLDM | 150 |
| NGTDM | 2 |

**Source:** https://pyradiomics.readthedocs.io/en/latest/features.html
